# Women’s thalassemia status and embryo carrier status do not affect pregnancy outcomes after euploid transfers

**DOI:** 10.3389/fendo.2025.1645590

**Published:** 2025-10-22

**Authors:** Li Fan, Zhetao Li, Wugao Li, Liuyan Wei, Ni Tang, Liuying Nong, Jingjing Li, Wenjie Huang

**Affiliations:** ^1^ Department of Reproductive Medicine, Guangzhou Women and Children’s Medical Center Liuzhou Hospital, Liuzhou, Guangxi, China; ^2^ Department of Reproductive Medicine, Liuzhou Maternity and Child Healthcare Hospital, Liuzhou, Guangxi, China; ^3^ Liuzhou Key Laboratory of Gynecologic Tumor, Liuzhou, Guangxi, China; ^4^ Guangxi Clinical Research Center for Obstetrics and Gynecology, Liuzhou, Guangxi, China; ^5^ Reproductive Genetics Department, The First Affiliated Hospital of Kunming Medical University, Kunming, Yunnan, China

**Keywords:** thalassemia, PGT-A, euploid embryo transfer, live birth rate, reproductive outcomes

## Abstract

**Background:**

While preimplantation genetic testing for monogenic disorders (PGT-M) is widely applied to prevent transmission of severe thalassemia, limited evidence exists regarding the impact of thalassemia carrier status or embryo genotype on pregnancy outcomes in patients undergoing preimplantation genetic testing for aneuploidy (PGT-A).

**Methods:**

This retrospective cohort study included 981 women who underwent their first autologous IVF cycle with single euploid embryo transfer at a tertiary reproductive center between January 2016 and December 2023. Among them, 512 were classified as having thalassemia (including heterozygous carriers and non–transfusion-dependent cases), and 460 were non-thalassemia controls. All participants underwent PGT-A, with a subset also receiving concurrent PGT-M. Clinical outcomes—including clinical pregnancy, live birth, and miscarriage rates—were compared between groups using Poisson regression models, adjusting for age, BMI, and endometrial thickness. Additional subgroup analyses stratified patients by age (<35 vs. ≥35 years) and embryo genotype (thalassemia carrier vs. non-carrier).

**Results:**

Thalassemia patients were significantly younger and had lower BMI and thicker endometrium compared to non-thalassemia patients. However, no statistically significant differences were found in clinical pregnancy (67.8% vs. 63.0%, aRR = 1.05, 95% CI: 0.94–1.16), live birth (61.9% vs. 53.3%, aRR = 1.08, 95% CI: 0.96–1.23), or miscarriage rates (5.9% vs. 9.8%, aRR = 0.82, 95% CI: 0.49–1.40) between the groups after adjustment. Furthermore, within the broadly defined thalassemia group, no significant differences were observed between embryos with and without pathogenic thalassemia variants. Interaction analyses showed no synergistic effects between thalassemia status and clinical variables.

**Conclusion:**

Thalassemia status and embryo genotype do not appear to adversely affect pregnancy outcomes following euploid embryo transfer. These findings support the transfer of heterozygous carrier embryos and suggest that individualized reproductive counseling and embryo selection strategies can be safely applied in couples at genetic risk of thalassemia undergoing IVF with PGT.

## Introduction

Thalassemia is a group of inherited hemoglobinopathies caused by impaired synthesis of globin chains, primarily classified into alpha (α) and beta (β) types. The severity of α-thalassemia depends on the number of gene deletions: Hb Bart’s hydrops fetalis (–/–) is typically lethal *in utero*, while HbH disease (–/-α) leads to mild to moderate anemia (1). β-thalassemia results from mutations in the HBB gene and is divided into major and intermedia forms. β-thalassemia major presents in infancy and requires lifelong transfusions and iron chelation, often complicated by iron overload. Intermedia cases are milder and may not require regular transfusions (2). With increased global migration, thalassemia has become a global public health issue, particularly in endemic regions such as the Mediterranean, Middle East, Southeast Asia, and southern China (3).

Iron overload, either from transfusions or ineffective erythropoiesis, often leads to pituitary iron deposition, suppressing LH and FSH secretion and impairing ovarian function. Clinically, this may result in amenorrhea or hypogonadism. Additionally, oxidative stress may damage the ovarian microenvironment, affecting oocyte quality and follicular development (4). Higher thalassemia carrier rates have been observed in infertile women, especially those with silent α-thalassemia, suggesting a potential link between carrier status and fertility (5). Although associations between thalassemia and female endocrine dysfunction have been reported (6, 7), a direct causal link to infertility remains unclear.

Preimplantation genetic testing for monogenic disorders (PGT-M) offers a valuable strategy for thalassemia carriers to avoid high-risk pregnancies. PGT-M enables detection of pathogenic variants at the embryonic stage, allowing selection of unaffected embryos (8). It has shown high diagnostic accuracy, even in complex cases lacking family samples, and significantly reduces the risk of having a child with severe thalassemia (9). Although not routinely paired with preimplantation genetic testing for aneuploidy (PGT-A), many patients choose to perform both tests concurrently during trophectoderm biopsy, as it adds no additional risk to the embryo. However, no studies have systematically evaluated pregnancy outcomes in thalassemia patients undergoing euploid embryo transfer, nor compared outcomes between euploid embryos with and without pathogenic thalassemia variants.

This study aims to: (1) compare clinical pregnancy, live birth, and miscarriage rates between women with and without thalassemia undergoing euploid embryo transfer, with subgroup analysis by age; and (2) assess whether thalassemia-related pathogenic variants in embryos affect outcomes in PGT-M plus PGT-A cycles. By analyzing 981 euploid embryo transfer cycles, this study seeks to clarify the influence of thalassemia status and carrier genotype on assisted reproductive outcomes, offering evidence to guide clinical decision-making in affected patients.

## Materials and methods

### Study design

This retrospective cohort study was approved by the Ethics Committee of Guangzhou Women and Children’s Medical Center, Liuzhou Hospital (No. 2024-238). The study included patients who underwent their first autologous *in vitro* fertilization (IVF) treatment at the center between January 2016 and December 2023. Eligible participants were women with thalassemia who underwent PGT-A and received a euploid embryo transfer, along with a control group of non-thalassemia patients treated during the same period. Exclusion criteria included cycles involving donor oocytes, donor sperm, or frozen/thawed oocytes, patients with anemia symptoms or other hereditary disorders, and cases in which the male partner of a non-thalassemia woman was diagnosed with thalassemia.

Because many patients were screened externally, detailed genotyping was not consistently available. Women were classified as having thalassemia if clinical or laboratory records indicated carrier or NTDT status. Women in the control group had no clinical or laboratory evidence of thalassemia; however, as comprehensive genetic screening was not systematically performed for all participants, the possibility of unrecognized carriers in the control group cannot be excluded.

### Ovarian stimulation protocols

All patients received individualized ovarian stimulation regimens based on their ovarian reserve and clinical characteristics. Common protocols included GnRH antagonist, long-acting or short-acting GnRH agonist protocols, and progestin-primed ovarian stimulation (PPOS). The choice of medications and initial gonadotropin doses were determined by patient age, baseline hormone levels, antral follicle count (AFC), and previous treatment responses. Follicular development was monitored via transvaginal ultrasound and serum hormone levels (E2, LH, and P). When criteria for ovulation triggering were met (e.g., ≥2 follicles measuring 17–18 mm or ≥3 follicles ≥16 mm), final oocyte maturation was induced with hCG, GnRH agonist, or both. Oocyte retrieval was performed 35–37 hours after trigger under transvaginal ultrasound guidance.

Intracytoplasmic sperm injection (ICSI) was the primary fertilization method unless otherwise specified by the patient. Embryos were morphologically assessed and graded on Day 1, Day 3, and Day 5. Day 5 blastocysts were graded according to the Istanbul consensus, based on the quality of the inner cell mass (ICM) and trophectoderm (TE), each scored as A (excellent), B (good), or C (poor). Blastocysts with both ICM and TE graded A or B (e.g., AA, AB, BA, BB) were classified as high-quality, while those with either component graded C (e.g., BC, CB) were classified as low-quality (10).

### PGT biopsy and vitrification–warming embryo transfer procedures

On Day 5 or 6 post-fertilization, blastocysts that reached the appropriate stage underwent PGT-A or combined PGT-M + PGT-A based on individualized indications. Prior to biopsy, an opening (15–20 μm) was made in the zona pellucida opposite the ICM using infrared laser. The embryos were cultured for an additional 4–6 hours to allow trophectoderm (TE) herniation. Under a micromanipulation system, 5–8 TE cells were aspirated and isolated using laser pulses, then transferred into PCR tubes containing lysis buffer for genetic analysis. After biopsy, embryos were cryopreserved by vitrification.

For PGT-A, whole genome amplification was performed using Berry Genomics PGS reagents, followed by high-throughput sequencing on the Illumina CN500 platform. For combined PGT-M + PGT-A, multiple displacement amplification (MDA) was followed by either STR-based linkage analysis with next-generation sequencing (NGS), or SNP array analysis integrated with BlueFuse software for concurrent pathogenic variant detection and chromosomal screening.

Upon confirmation of embryo transfer eligibility, endometrial preparation was conducted using either natural or artificial cycles, based on physician and patient preference. Embryo transfer was scheduled when endometrial conditions were optimal, followed by routine luteal phase support and close monitoring of pregnancy outcomes.

### Outcomes

The primary outcome was live birth, defined as the delivery of at least one live-born infant. Secondary outcomes included clinical pregnancy and miscarriage. Clinical pregnancy was defined as ultrasound confirmation of an intrauterine gestational sac after euploid embryo transfer; biochemical pregnancies were not counted as clinical pregnancies. Miscarriage was defined as the spontaneous loss of an intrauterine clinical pregnancy before 20 gestational weeks. Ectopic pregnancies were documented and analyzed separately.

### Statistical analysis

Continuous variables, which did not follow a normal distribution, were summarized as median and interquartile range (IQR). No missing data were observed for outcome variables, and missing covariate data were <2%. Median imputation was applied to minimize outlier impact and improve data stability.

For group comparisons, the Mann–Whitney U test was used for continuous variables, and the chi-square test for categorical variables. To evaluate differences in pregnancy outcomes following euploid embryo transfer between women with and without thalassemia, Poisson regression models were constructed. Results were expressed as relative risk (RR) with 95% confidence intervals (CIs), adjusting for age, body mass index (BMI), and endometrial thickness. To explore interactions between thalassemia status and clinical variables, interaction terms (e.g., group × variable) were added to the regression models. Main effects of the interacting covariate were excluded from the models to avoid redundancy. Robust standard errors were used to address heteroscedasticity. Interaction results were reported as estimated RRs and corresponding 95% CIs. All analyses were performed using SPSS version 26.0 and RStudio. A two-sided p-value < 0.05 was considered statistically significant.

## Results

A total of 981 women who underwent euploid embryo transfer were included in this study. Patients were categorized into two groups based on thalassemia status: the non-thalassemia group (n = 460) and the thalassemia group (n = 512). The median age of patients in the non-thalassemia group was 38 years (IQR: 34–40), significantly higher than that of the thalassemia group, which was 32 years (IQR: 29–35) (p < 0.001). Age distribution further revealed that 71.7% of patients in the thalassemia group were under 35 years old, compared to only 26.3% in the non-thalassemia group. Conversely, a higher proportion of patients aged 38 years or older was observed in the non-thalassemia group (55.4% vs. 13.1%). In terms of BMI, the median BMI in the thalassemia group was 21.50 (IQR: 19.90–23.32), significantly lower than that in the non-thalassemia group (22.20, IQR: 20.44–24.44) (p < 0.001). A higher proportion of patients with BMI < 18.5 was noted in the thalassemia group (10.7% vs. 7.4%), while the proportion of patients with BMI ≥ 25 was lower. Additionally, the median endometrial thickness was significantly greater in the thalassemia group (10.0 mm vs. 9.2 mm, p < 0.001). There were no significant differences between the two groups regarding the number of embryos transferred (median = 1), embryo quality (approximately 70% high-quality embryos), or endometrial preparation methods (**Table 1**).

**Table 1:** Demographic and baseline clinical characteristics of women with and without thalassemia undergoing euploid embryo transfer.

Characteristics	Non-thalassemia (n = 460)	Thalassemia (n = 512)	p value
Age, y			
Overall, median (IQR)	38 (34 - 40)	32 (29 - 35)	<.001
Age group, No. (%)			
< 35	121 (26.3)	367 (71.7)	
35-37	84 (18.3)	78 (15.2)	
38-40	155 (33.7)	51 (10)	
41-42	76 (16.5)	11 (2.1)	
> 42	24 (5.2)	5 (1)	
BMI			<.001
Overall, median (IQR)	22.20 (20.44 - 24.44)	21.50 (19.90 - 23.32)	
Category, No. (%)			
< 18.5	34 (7.4)	55 (10.7)	
18.5-24.9	333 (72.4)	393 (76.8)	
25-29.9	84 (18.3)	58 (11.3)	
≥ 30	9 (1.9)	6 (1.2)	
Endometrial thickness	9.2 (8.4 - 10.93)	10 (9 - 11.3)	<.001
No. embryos transferred	1 (1 - 1)	1 (1 - 1)	.503
Embryo quality^a^			.176
Low-quality	116 (25.2)	150 (29.3)	
Good-quality	344 (74.8)	362 (70.7)	
Endometrial preparation, No. (%)			0.757
Natural cycle	119 (25.9)	138 (27)	
Programmed cycle	341 (74.1)	374 (73)	

IQR, interquartile range; BMI, body mass index (calculated as weight in kilograms divided by height in meters squared). ^a^ Embryo quality: All biopsied embryos were blastocysts; grades AA, AB, BA, and BB were classified as good-quality embryos, whereas BC and CB were classified as low-quality embryos.

The clinical pregnancy rate was 67.8% in the thalassemia group and 63.0% in the non-thalassemia group, with no statistically significant difference (RR = 1.08, 95% CI: 0.98–1.18; aRR = 1.05, 95% CI: 0.94–1.16). The live birth rate was 61.9% in the thalassemia group and 53.3% in the non-thalassemia group. While this difference was significant in the unadjusted model (RR = 1.16, 95% CI: 1.04–1.30), it lost statistical significance after adjusting for age, BMI, and endometrial thickness (aRR = 1.08, 95% CI: 0.96–1.23). The miscarriage rate was lower in the thalassemia group (5.9% vs. 9.8%) with a significant unadjusted difference (RR = 0.60, 95% CI: 0.38–0.93), but the difference became non-significant after adjustment (aRR = 0.82, 95% CI: 0.49–1.40) (**Table 2**).

**Table 2:** Comparison of clinical pregnancy, live birth, and miscarriage outcomes after euploid embryo transfer between women with and without thalassemia.

Outcomes	Event. No./total (%)	Absolute difference, % (95% Cl)	Relative risk (95% Cl)	
			Unadjusted	Adjusted^a^
Clinical pregnancy				
Non-thalassemia	290/460 (63.0)	Reference	1 [Reference]	1 [Reference]
Thalassemia	347/512 (67.8)	4.8 (-1.2 to 10.8)	1.08 (0.98 - 1.18)	1.05 (0.94 - 1.16)
Live birth				
Non-thalassemia	245/460 (53.3)	Reference	1 [Reference]	1 [Reference]
Thalassemia	317/512 (61.9)	8.6 (2.4 to 14.8)	1.16 (1.04 - 1.3)	1.08 (0.96 - 1.23)
Miscarriage				
Non-thalassemia	45/460 (9.8)	Reference	1 [Reference]	1 [Reference]
Thalassemia	30/512 (5.9)	-3.9 (-7.3 to -0.5)	0.6 (0.38 - 0.93)	0.82 (0.49 - 1.4)

CI, confidence interval.

a Adjusted for age, BMI and endometrial thickness.

To explore potential synergistic effects, we conducted interaction analyses between thalassemia status and other baseline characteristics. No significant interactions were observed between thalassemia and age, BMI, endometrial thickness, embryo quality, or endometrial preparation method for clinical pregnancy, live birth, or miscarriage outcomes (all p > 0.05), indicating no evident synergistic effects (**Supplementary Table S1**).

To further investigate potential differences in live birth outcomes, we analyzed the “conditional live birth rate,” defined as the proportion of live births among those with clinical pregnancies. The rate was significantly higher in the thalassemia group (91.4%) compared to the non-thalassemia group (84.5%). The unadjusted relative risk was 1.08 (95% CI: 1.02–1.15), but the adjusted analysis showed no significant difference (aRR = 1.04, 95% CI: 0.97–1.11).

Given the significant age distribution difference between groups, we performed a stratified analysis by age (<35 years and ≥35 years) to compare pregnancy outcomes. Among women <35 years old, the live birth rate was higher in the thalassemia group (63.8% vs. 52.9%), with a statistically significant difference in the unadjusted model (RR = 1.21, 95% CI: 1.002–1.45), though the difference was not significant after adjustment (aRR = 1.14, 95% CI: 0.95–1.37). Clinical pregnancy and miscarriage rates did not differ significantly between the two groups in this subgroup. Among women ≥35 years old, no significant differences were observed in clinical pregnancy rate (67.6% vs. 64.0%), live birth rate (57.2% vs. 53.4%), or miscarriage rate (10.3% vs. 10.6%) between the thalassemia and non-thalassemia groups (**Supplementary Tables S2**, **S3**).

To examine whether embryo thalassemia mutation status influenced pregnancy outcomes, we compared outcomes of euploid embryos with and without thalassemia mutations among women with thalassemia. There were no significant differences in baseline characteristics between the two groups, including age, BMI, endometrial thickness, embryo quality, and endometrial preparation method (**Supplementary Table S4**), indicating overall comparability. In terms of pregnancy outcomes, the clinical pregnancy rate was similar between embryos with mutations and those without (68.2% vs. 67.2%, aRR = 0.99, 95% CI: 0.87–1.11), as was the live birth rate (62.5% vs. 61.1%, aRR = 0.98, 95% CI: 0.85–1.12). Miscarriage rates were also comparable (5.7% vs. 6.1%, aRR = 1.06, 95% CI: 0.54–2.09) (**Table 3**).

**Table 3:** Comparison of clinical pregnancy, live birth, and miscarriage outcomes between thalassemia mutation-carrying euploid embryos and normal euploid embryos in women with thalassemia.

Outcomes	Event. No./total (%)	Absolute difference, % (95% Cl)	Relative risk (95% Cl)	
			Unadjusted	Adjusted^a^
Clinical pregnancy				
Thalassemia	193/283 (68.2)	Reference	1 [Reference]	1 [Reference]
Normal	154/229 (67.2)	-1 (-9.5 to 7.5)	0.99 (0.87-1.11)	0.99 (0.87-1.11)
Live birth				
Thalassemia	177/283 (62.5)	Reference	1 [Reference]	1 [Reference]
Normal	140/229 (61.1)	-1.4 (-10.2 to 7.4)	0.98 (0.85-1.12)	0.98 (0.85-1.12)
Miscarriage				
Thalassemia	16/283 (5.7)	Reference	1 [Reference]	1 [Reference]
Normal	14/229 (6.1)	0.4 (-3.9 to 4.7)	1.08 (0.54-2.17)	1.06 (0.54-2.09)

CI, confidence interval.

**^a^** Adjusted for age, BMI and endometrial thickness.

## Discussion

In this study, we found that although women with thalassemia exhibited more favorable baseline reproductive characteristics—particularly in terms of age, BMI, and endometrial thickness—compared to those without thalassemia, no statistically significant differences were observed between the two groups in clinical pregnancy rate, live birth rate, or miscarriage rate after euploid embryo transfer, once relevant confounders were adjusted for. These findings indicate that thalassemia status alone may not independently influence pregnancy outcomes following PGT-A. Based on this evidence, women with thalassemia can expect reproductive outcomes comparable to the general population within the PGT-A-assisted reproductive pathway.

Our findings are largely consistent with previous studies and provide important complementary insights. For instance, Mensi et al. compared oocyte and embryo development parameters between women with transfusion-dependent thalassemia (TDT) and controls undergoing IVF. Despite lower AMH and AFC levels, fertilization and cleavage rates were slightly higher in the TDT group, with a comparable proportion of high-quality embryos (11). However, that study had a limited sample size and primarily focused on oocyte developmental potential. Pregnancy outcomes, though reported in **Supplementary Tables** (clinical pregnancy rate 33% vs. 35%; live birth rate 19% vs. 32%), were not primary endpoints and lacked statistical adjustment for potential confounders. In our recent larger-scale retrospective study, we compared reproductive outcomes between patients with NTDT and thalassemia carriers (TC), finding no significant differences in pregnancy outcomes across both fresh and frozen embryo transfer cycles (12). Nevertheless, since the control group consisted of genetic thalassemia carriers, the external validity of those findings may be limited. In contrast, the current study included 981 PGT-A-based euploid embryo transfer cycles, with standardized embryo screening and laboratory protocols, effectively minimizing variability in embryo quality.

Women with thalassemia undergoing PGT-A represent a unique population. Unlike traditional PGT-A candidates, who typically present with infertility, advanced maternal age, or recurrent pregnancy loss, many in this cohort actively sought assisted reproduction for monogenic disease screening despite the absence of infertility. This likely contributes to their favorable BMI and endometrial characteristics. Prior studies have demonstrated that younger maternal age, lower BMI, and optimal endometrial conditions are positively associated with pregnancy success (13–15).

Age-stratified analysis revealed that among women under 35, live birth rates were slightly higher in the thalassemia group; however, these differences were not statistically significant after adjustment. Similarly, no significant outcome differences were observed among women aged 35 and above. Interaction analyses further confirmed that thalassemia status did not exert any synergistic effects with baseline characteristics on pregnancy outcomes. Therefore, the observed favorable outcomes in this group are more likely attributable to high-quality embryo selection and standardized clinical protocols provided by PGT-A, rather than the intrinsic physiological or pathological features associated with thalassemia.

Notably, our study also demonstrated that euploid embryos carrying pathogenic thalassemia mutations did not show poorer pregnancy outcomes. This aligns with the current consensus in the PGT-M field, which holds that phenotypically normal heterozygous embryos for autosomal recessive disorders are acceptable candidates for transfer (16). Clinically, some patients—particularly those with previous IVF failures—express concern about the potential reproductive risks associated with thalassemia, especially the impact of iron overload on implantation (17). Recent studies have suggested that iron overload may lead to mitochondrial dysfunction via reactive oxygen species (ROS), disrupting microtubule structures and impairing spindle function, thereby increasing the risk of chromosomal segregation errors during oocyte maturation (18, 19). However, our clinical findings did not support a decline in implantation potential for embryos carrying pathogenic mutations, offering real-world evidence to counter these theoretical concerns.

To our knowledge, this study represents the largest cohort to date evaluating pregnancy outcomes in women with thalassemia undergoing PGT-A. In clinical practice, couples in which only one partner is a thalassemia carrier or presents with mild symptoms are often managed through conventional IVF without PGT-M, reflecting a generally favorable view of their reproductive prognosis. However, robust data on their outcomes following PGT-A remain limited, restricting evidence-based counseling and individualized management strategies. Notably, all embryo biopsies and genetic testing in our study were conducted in a single laboratory, reducing technical variability and enhancing internal consistency. Despite these strengths, several limitations should be acknowledged. As a single-center retrospective study, it is subject to potential bias and missing data. The single-site nature of the cohort and laboratory also limits external generalizability. Moreover, although we evaluated pregnancy outcomes up to live birth, postnatal data—such as neonatal birth weight, Apgar scores, and other perinatal indicators—were not available, precluding conclusions about long-term child health outcomes. In addition, because not all participants underwent standardized genetic screening, some thalassemia carriers may have been inadvertently included in the control group, which could attenuate the observed differences between groups.

## Conclusion

In conclusion, our study suggests that thalassemia status does not independently affect pregnancy outcomes following euploid embryo transfer. Clinicians may offer reproductive prognoses and counseling strategies to patients with mild or heterozygous thalassemia that are comparable to those for the general population. Importantly, euploid embryos carrying pathogenic thalassemia variants but without phenotypic abnormalities also demonstrate favorable reproductive potential, providing valuable guidance for embryo selection and clinical decision-making.

## Data Availability

The original contributions presented in the study are included in the article/**Supplementary Material**. Further inquiries can be directed to the corresponding author.
