# Supplementary material for: Women’s thalassemia status and embryo carrier status do not affect pregnancy outcomes after euploid transfers
Source: Front Endocrinol (Lausanne). 2025 Oct 22;16:1645590. doi: 10.3389/fendo.2025.1645590 (PMC12586123; doi:10.3389/fendo.2025.1645590)
Supplement: Supplementary file 1 [file Table1.docx]

**Supplemental table 1.** Interaction Analysis of Thalassemia Status With Age, BMI, Endometrial Thickness, Embryo Quality, and Endometrial Preparation on Clinical Pregnancy, Live Birth, and Miscarriage Outcomes.

| **Interaction** | **Relative risk (95% Cl)** | **p value** |
| --- | --- | --- |
| Clinical pregnancy | | |
| Thalassemia: Age | 0.99 (0.97-1.02) | 0.553 |
| Thalassemia: BMI | 1.01 (0.98-1.04) | 0.5376 |
| Thalassemia: Endometrial thickness | 0.97 (0.93-1.02) | 0.2353 |
| Thalassemia: Embryo quality | 1.11 (0.86-1.43) | 0.4125 |
| Thalassemia: Endometrial preparation | 1.01 (0.83-1.23) | 0.9523 |
| Live birth | | |
| Thalassemia: Age | 0.99 (0.96-1.01) | 0.4049 |
| Thalassemia: BMI | 1.02 (0.98-1.06) | 0.2701 |
| Thalassemia: Endometrial thickness | 0.97 (0.92-1.02) | 0.2056 |
| Thalassemia: Embryo quality | 1.01 (0.74-1.36) | 0.9637 |
| Thalassemia: Endometrial preparation | 1.09 (0.87-1.37) | 0.452 |
| Miscarriage | | |
| Thalassemia: Age | 1.06 (0.96-1.16) | 0.2545 |
| Thalassemia: BMI | 0.99 (0.85-1.15) | 0.8538 |
| Thalassemia: Endometrial thickness | 0.93 (0.77-1.13) | 0.4816 |
| Thalassemia: Embryo quality | 1.36 (0.52-3.51) | 0.5296 |
| Thalassemia: Endometrial preparation | 0.67 (0.22-2.07) | 0.488 |

**Supplemental table 2.** Baseline Characteristics of Non-Thalassemia and Thalassemia Women Undergoing Euploid Embryo Transfer, Stratified by Age (< 35 vs ≥35 Years).

| Characteristics | Age < 35 | | | Age ≥ 35 | | |
| --- | --- | --- | --- | --- | --- | --- |
|  | Non-thalassemia  (n = 121) | Thalassemia  (n = 367) | p | Non-thalassemia  (n = 339) | Thalassemia  (n = 145) | p |
| Age, y |  |  |  |  |  |  |
| Overall, median (IQR) | 32 (29 - 33) | 30 (28 - 32) | < .001 | 39 (38 - 41) | 37 (35 - 39) | < .001 |
| BMI |  |  | .32 |  |  | .33 |
| Overall, median (IQR) | 21.48 (19.61 - 23.19) | 21.20 (19.56 - 22.82) |  | 22.51 (20.81 - 24.77) | 22.22 (20.81 - 24.34) |  |
| Category, No. (%) |  |  |  |  |  |  |
| < 18.5 | 13 (10.7) | 47 (12.8) |  | 21 (6.2) | 8 (5.5) |  |
| 18.5-24.9 | 90 (74.4) | 284 (77.4) |  | 243 (71.7) | 109 (75.2) |  |
| 25-29.9 | 16 (13.2) | 31 (8.4) |  | 68 (20.1) | 27 (18.6) |  |
| ≥ 30 | 2 (1.7) | 5 (1.4) |  | 7 (2.1) | 1 (0.7) |  |
| Endometrial thickness | 9 (8 - 10.6) | 10 (9 - 11.5) | < .001 | 9.2 (8.5 - 11) | 9.8 (8.8 - 11) | .052 |
| No. embryos transferred | 1 (1 - 1) | 1 (1 - 1) | .57 | 1 (1 - 1) | 1 (1 - 1) | .36 |
| Embryo quality |  |  | .17 |  |  | .30 |
| Low-quality | 26 (21.5) | 104 (28.3) |  | 90 (26.5) | 46 (31.7) |  |
| Good-quality | 95 (78.5) | 263 (71.7) |  | 249 (73.5) | 99 (68.3) |  |
| Endometrial preparation, No. (%) | |  | .81 |  |  | .03 |
| Natural cycle | 30 (24.8) | 85 (23.2) |  | 89 (26.3) | 53 (36.6) |  |
| Programmed cycle | 91 (75.2) | 282 (76.8) |  | 250 (73.7) | 92 (63.4) |  |

**Supplemental table 3.** Comparison of Clinical Pregnancy, Live Birth, and Miscarriage Outcomes Between Women With and Without Thalassemia Undergoing Euploid Embryo Transfer, Stratified by Age (< 35 vs ≥35 Years).

| < 35 |  |  | |
| --- | --- | --- | --- |
| outcomes | Event. No./total (%) | Relative risk (95% Cl) | |
|  |  | unadjusted | adjusted^a^ |
| clinical pregnancy |  |  |  |
| control | 73/121 (60.3) | 1 [Reference] | 1 [Reference] |
| thalassemia | 249/367 (67.8) | 1.12 (0.96-1.32) | 1.08 (0.92-1.27) |
| Live birth |  |  |  |
| control | 64/121 (52.9) | 1 [Reference] | 1 [Reference] |
| thalassemia | 234/367 (63.8) | 1.21 (1.002-1.45) | 1.14 (0.95-1.37) |
| Miscarriage |  |  |  |
| control | 9/121 (7.4) | 1 [Reference] | 1 [Reference] |
| thalassemia | 15/367 (4.1) | 0.55 (0.25-1.22) | 0.7 (0.31-1.56) |
| ≥ 35 |  |  |  |
| clinical pregnancy |  |  |  |
| control | 217/339 (64) | 1 [Reference] | 1 [Reference] |
| thalassemia | 98/145 (67.6) | 1.06 (0.92-1.21) | 1.02 (0.88-1.18) |
| Live birth |  |  |  |
| control | 181/339 (53.4) | 1 [Reference] | 1 [Reference] |
| thalassemia | 83/145 (57.2) | 1.07 (0.9-1.27) | 1.03 (0.86-1.24) |
| Miscarriage |  |  |  |
| control | 36/339 (10.6) | 1 [Reference] | 1 [Reference] |
| thalassemia | 15/145 (10.3) | 0.97 (0.55-1.72) | 0.96 (0.53-1.73) |

^a^ Adjusted for age, BMI, endometrial thickness and Endometrial preparation.

**Supplemental table 4.** Baseline characteristics of women with thalassemia: comparison between euploid embryos carrying thalassemia mutations and normal euploid embryos.

| Characteristics | Thalassemia embryos  (n = 283) | Normal embryos  (n = 229) | p value |
| --- | --- | --- | --- |
| Age, y |  |  |  |
| Overall, median (IQR) | 32 (29 - 35) | 32 (29 - 35) | .41 |
| Age group, No. (%) |  |  |  |
| < 35 | 208 (73.5) | 159 (69.4) |  |
| 35-37 | 37 (13.1) | 41 (17.9) |  |
| 38-40 | 28 (9.9) | 23 (10) |  |
| 41-42 | 6 (2.1) | 5 (2.2) |  |
| > 42 | 4 (1.4) | 1 (0.4) |  |
| BMI |  |  | .43 |
| Overall, median (IQR) | 21.63 (19.92 - 23.38) | 21.33 (19.81 - 23.24) |  |
| Category, No. (%) |  |  |  |
| < 18.5 | 27 (9.5) | 28 (12.2) |  |
| 18.5-24.9 | 219 (77.4) | 174 (76) |  |
| 25-29.9 | 33 (11.7) | 25 (10.9) |  |
| ≥ 30 | 4 (1.4) | 2 (0.9) |  |
| Endometrial thickness | 10 (9 - 11.5) | 10 (9 - 11) | .86 |
| No. embryos transferred | 1 (1 - 1) | 1 (1 - 1) | .37 |
| Embryo quality |  |  | .78 |
| Low-quality | 81 (28.6) | 69 (30.1) |  |
| Good-quality | 202 (71.4) | 160 (69.9) |  |
| Endometrial preparation, No. (%) | |  | .34 |
| Natural cycle | 71 (25.1) | 67 (29.3) |  |
| Programmed cycle | 212 (74.9) | 162 (70.7) |  |
